# *MiR-10* Represses *HoxB1a* and *HoxB3a* in Zebrafish

**DOI:** 10.1371/journal.pone.0001396

**Published:** 2008-01-02

**Authors:** Joost M. Woltering, Antony J. Durston

**Affiliations:** Institute of Biology, Leiden University, Leiden, The Netherlands; University of Washington, United States of America

## Abstract

**Background:**

The *Hox* genes are involved in patterning the anterior-posterior axis. In addition to the protein coding *Hox* genes, the *miR-10*, *miR-196* and *miR-615* families of microRNA genes are conserved within the vertebrate *Hox* clusters. The members of the *miR-10* family are located at positions associated with *Hox-4* paralogues. No function is yet known for this microRNA family but the genomic positions of its members suggest a role in anterior-posterior patterning.

**Methodology/Principal Findings:**

Using sensor constructs, overexpression and morpholino knockdown, we show in Zebrafish that *miR-10* targets *HoxB1a* and *HoxB3a* and synergizes with *HoxB4* in the repression of these target genes. Overexpression of *miR-10* also induces specific phenotypes related to the loss of function of these targets. *HoxB1a* and *HoxB3a* have a dominant hindbrain expression domain anterior to that of *miR-10* but overlap in a weaker expression domain in the spinal cord. In this latter domain, *miR-10* knockdown results in upregulation of the target genes. In the case of a *HoxB3a* splice variant that includes *miR-10c* within its primary transcript, we show that the microRNA acts in an autoregulatory fashion.

**Conclusions/Significance:**

We find that *miR-10* acts to repress *HoxB1a* and *HoxB3a* within the spinal cord and show that this repression works cooperatively with *HoxB4*. As with the previously described interactions between *miR-196* and *HoxA7* and *Hox-8* paralogues, the target genes are located in close proximity to the microRNA. We present a model in which we postulate a link between the clustering of *Hox* genes and post-transcriptional gene regulation. We speculate that the high density of transcription units and enhancers within the *Hox* clusters places constraints on the precision of the transcriptional control that can be achieved within these clusters and requires the involvement of post-transcriptional gene silencing to define functional domains of genes appropriately.

## Introduction

*Hox* genes participate in regionalizing the anterior-posterior body axis in metazoan animals. In mammals, this gene family comprises 39 closely related genes for homeodomain transcription factors, organized in 4 homologous clusters (A, B, C, D) [1]–[4]. The genes are expressed along the body axis in a sequence that corresponds to their genomic sequence within the *Hox* clusters. The more 3′ a gene is located in a *Hox* cluster the more anterior is its expression domain. This feature is commonly referred to as ‘spatial colinearity’. The *Hox* genes have sharply defined anterior expression boundaries but their posterior boundaries are typically less clear and overlap with the expression of more posterior *Hox* genes.

The 4 mammalian *Hox* clusters arose from an ancestral *Hox* cluster via two genome duplications. In *Teleost* fish, an additional genome duplication generated 8 *Hox* clusters (named *Aa*, *Ab*, etc.), there being 7 clusters containing 49 genes in Zebrafish [5], [6], [7] due to loss of one cluster during evolution.

In addition to the *Hox* coding genes, the *miR-10*, *miR-196* and *miR-615* microRNA gene families have been identified within the vertebrate *Hox* clusters [8]–[11]. MicroRNAs are small (∼22 nt) non-coding RNAs which are derived from stemloop forming precursor transcripts, through processing by the RNAse III enzymes *Dicer* [12] and *Drosha* [13]. MicroRNAs function in post-transcriptional gene silencing by binding to imperfect target sites in messengerRNAs. They thereby induce translational inhibition and RNA destabilization [14], [15].

In the *Hox* clusters, *miR-10* genes are closely associated with the positions of *Hox-4* paralogue members, *miR-196* is located 5′ of *Hox-9* paralogues and the more recently cloned *miR-615* is located in the *HoxC5* intron in mammals but appears to be absent from *Teleosts* and *Xenopus tropicalis*. The latter microRNA could therefore be restricted either to mammals or to amniotes. In the Zebrafish genome, *miR-10* is present in 5 paralogues representing 4 different isoforms (*a*, *b*, *c* and *d*), which differ from each other at 1 to 3 positions [16], [17]. We previously showed that the genomic location of the *miR-10d* microRNA corresponds to the degenerated *HoxDb* cluster [16].

Mouse knockouts and a Zebrafish germline *Dicer* mutant have revealed important functions for microRNAs in the coordination of normal embryonic development [18], [19], but the individual vertebrate microRNAs are in general still enigmatic genetic objects and only a few have been characterized at a functional level. In Zebrafish, *miR-430* [20] has been shown to silence maternal RNAs, *miR-214* is involved in proper somite specification [21] and *miR-375* is necessary for the maintenance of embryonic pancreas integrity [22].

With respect to the *Hox* related microRNAs, *HoxA7* and *Hox-8* paralogues have been identified as targets of *miR-196* [23], [24], [25]. In chicken the interaction with *HoxB8* has been implicated in the mechanism that abolishes the competence of posterior lateral plate mesoderm for limb induction by retinoic acid [25]. In *Drosophila*, a conserved or possibly convergent interaction exists for the *miR-196* homologue *IAB-4* which targets the *Ubx Hox* gene [26]. Until now, the function of the *miR-10* microRNA family has remained unclear but, based on its evolutionary conservation within the anterior part of the *Hox* clusters, an associated role in anterior-posterior patterning seems likely.

The anterior *Hox* genes are strongly expressed in the central nervous system and play an important role in patterning the hindbrain, spinal cord and branchial arches [27], [28], [29]. In the hindbrain, the expression of these *Hox* genes follows the rhombomeric boundaries. *HoxB1a* and *HoxB1b* are expressed in rhombomere (r) 4, *HoxB2a* defies the rule of colinearity and is expressed more anteriorly, in r 3 and r 4, *HoxB3a* and *HoxA3a* are expressed most strongly in r 5 and 6 with a weaker domain extending more posteriorly in the spinal cord. The *Hox-4* paralogues are expressed from r 7 onwards and throughout the spinal cord (reviewed 30). The pattern of *Hox* expression in the hindbrain contributes to the formation of localized neuronal structures like the rhombomere 4 specific Mauthner neurons and the distinct patterns of cranial motor nerves in different regions of the hindbrain.

Here, we address the function of *miR-10* with relation to a possible role in anterior-posterior patterning. We show that *miR-10* represses the nearby *HoxB1a* and *HoxB3a* genes and that its overexpression also induces the associated loss of function phenotypes for both genes. *MiR-10* morphant embryos show upregulation of these target genes within the normal *miR-10* expression domain, indicating that active repression occurs in the embryo. In overexpression experiments, *miR-10* synergizes with *HoxB4* in the repression of these target genes. In the case of a long range *HoxB3a* transcript that includes *miR-10c* within its primary transcript, we show that the microRNA acts in an essentially auto regulatory fashion. In addition, we present a model in which we explain the need for post-transcriptional regulatory interactions within the *Hox* clusters on basis of the high density of enhancers and transcription units within the clusters.

## Results

### MiR-10 is expressed in a Hox-4 like pattern

**Figure 1:**
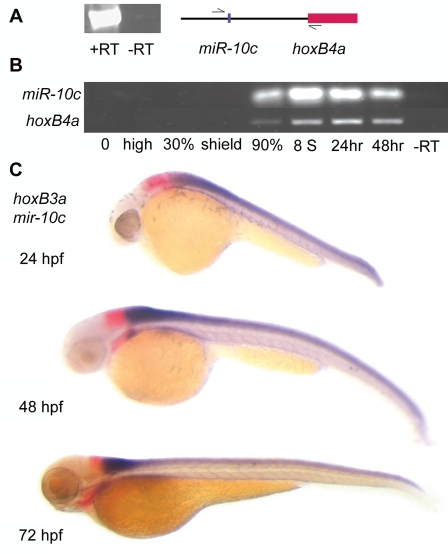
Spatial and temporal expression profile of *miR-10c.* A) RT-PCR with primers located 5′ of *miR-10c* and within the coding region of exon 1 of *HoxB4a* shows inclusion of *miR-10c* and *HoxB4a* on the same transcript. PCR 35 cycles, -RT: no reverse transcriptase added. B) RT-PCR shows similar temporal expression during development of *HoxB4a* (28 cycles) and *miR-10c* pre-miRNA (35 cycles). C) Whole mount *in situ* hybridization on different stage Zebrafish embryos shows mutually exclusive expression of the *HoxB3a* rhombomere 5/6 domain (red) with *miR-10c* (purple).

The *MiR-10* paralogues are associated with the 5′ genomic region of *Hox-4* genes and microRNA specific Locked Nucleic Acid (LNA) *in situ* hybridization [16], [31] and transgenic sensor lines [24] have revealed similar patterns of expression as for the *Hox-4* paralogues. RT-PCR with primers located 5′ of *miR-10c* and in the *HoxB4a* coding sequence shows that *miR-10c* and *HoxB4a* are located on the same primary transcript (figure 1A) and RT-PCR for the individual genes shows the same temporal expression pattern (figure 1B).

The exact anterior boundary of the neural expression of *miR-10c* was determined in double *in situ* hybridization together with the anterior neighboring gene *HoxB3a* (figure 1C). Consistent with the transcriptionally implied co-regulation, *miR-10c* has the same anterior boundary of expression as described for *HoxB4a* and is expressed in a mutually exclusive domain with the anterior strong r 5/6 expression domain of *HoxB3a* (single *in situ*, figure S1A).

Under some circumstances, LNA probes are known to exhibit single nucleotide resolution [16], [32]. *In situ* hybridization with probes matching each of the *miR-10* isoforms (figure S2) excludes that other *miR-10* isoforms, which are possibly not detected by the *miR-10c* LNA probe, are expressed in domains overlapping with or anterior to the main expression domain of *HoxB3a*. The expression patterns of the *miR-10* isoforms differ in that the probes for *miR-10b* and *miR-10d* show a more posterior rostral boundary, with highest intensity staining caudal to the hindbrain, while *miR-10a* and *miR-10c* probes have an anterior boundary at r6/7.

### MiR-10 target sites are present in HoxB1a and HoxB3a

**Figure 2:**
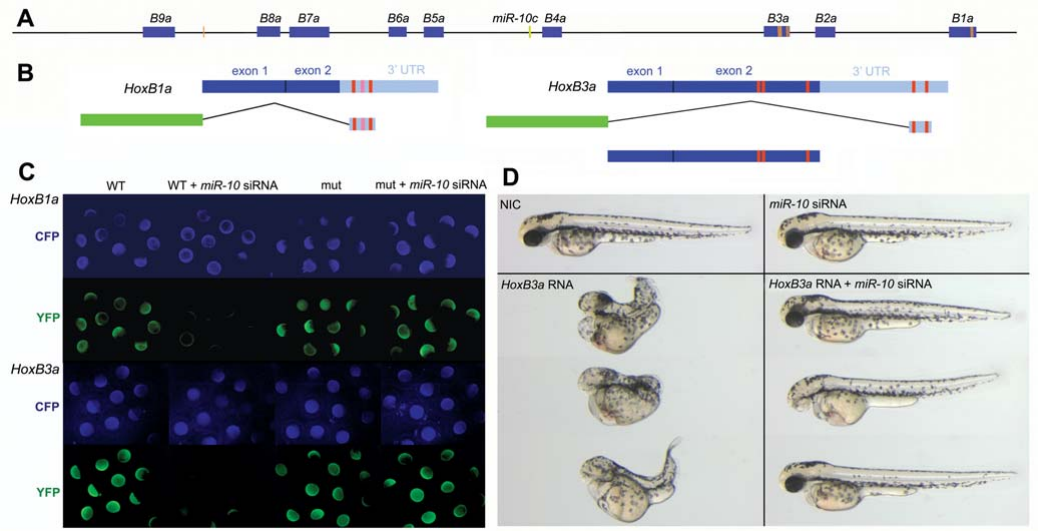
* miR-10* target sites within the *HoxBa* cluster. A) Schematic representation of the zebrafish *HoxBa* cluster with *MiR-10* seed sequences (nucleotide 1-7) within the sense strand indicated as orange bars. Known and EST database inferred mature *Hox* transcripts are indicated in blue. The *miR-10c* microRNA gene is indicated in green. B) Schematic representation of the *HoxB1a* and *HoxB3a E-YFP* sensor constructs and the *HoxB3a* overexpression construct. Red boxes indicate the position of the seed sequences. The light red box in the *HoxB1a* 3′ UTR is a target site flanked at position one by a T instead of an A. C) Validation of the *HoxB1a* and *HoxB3a E-YFP* sensor constructs by injection of wildtype (WT) and seed mutant (mut) constructs in presence and absence of *miR-10* siRNA. *E-CFP* was co-injected as a loading control. D) Phenotypic sensor assay to validate the *HoxB3a* ORF *miR-10* target sites. Overexpression of 40pg *HoxB3a* results in severe anterior and posterior truncations that are rescued by co-injection with *miR-10* siRNA

MicroRNAs bind through a complementary fuzzy match to target sites in messengerRNAs. The specificity of this interaction resides in the sequence of nucleotides 2-7 of the microRNA, called ‘the seed’, which does not differ between the different isoforms of a microRNA family. This sequence forms the minimal requirement for a target site and is usually flanked at position 1 by an adenosine or a perfect match [33]. Accordingly, sequence information permits the prediction of target genes. *In silico* target analysis in *Teleosts* has predicted the presence of *miR-10* target sites within the *Hox* clusters [34]. Inspection of the Zebrafish *HoxBa* cluster with the *miR-10* seed sequence (nucleotide1-7) indicates the presence of putative target sites primarily in the 3′ part of the cluster. Candidate target sequences are associated with two *Hox* transcripts: 2 sites are located in the 3′ UTR of *HoxB1a*, 2 sites in the 3′ UTR of *HoxB3a* and 3 sites are present in the *HoxB3a* open reading frame (figure 2A).

*E-YFP* sensor constructs containing the *HoxB1a* and *HoxB3a* 3′UTR target sequences were tested for their sensitivity to silencing by *miR-10* (figure 2B). Constructs containing point mutations in the seed sequence were used as negative controls. Sensor construct RNA was injected with or without *miR-10* siRNA. Co-injection with *E-CFP* RNA was used as a loading control. Embryos were analyzed for fluorescence at blastula/early gastrula stages. Both *HoxB1a* and *HoxB3a* wildtype sensor constructs are strongly repressed by the microRNA (figure 2C), while the seed point mutant construct proves insensitive to repression by *miR-10*. The seed mutant construct for *HoxB1a* in which the two target sites are mutated is still partially silenced however after co-injection with *miR-10* (data not shown). Closer inspection revealed a 3^rd^ possible target sequence corresponding to nucleotide 2-7 flanked by a T at position 1. After introduction of a point mutation into this seed sequence the construct is no longer repressed by injection of *miR-10 (* figure 2C).

A phenotypic sensor assay was used to validate the target sites located in the *HoxB3a* ORF. Overexpression of 40pg *HoxB3a* RNA induces a very strong phenotype with both anterior and posterior truncations of the embryo (figure 2D). These defects are completely rescued by co-injection of *miR-10* siRNA, indicating absence of overexpressed *HoxB3a* protein. These experiments identify the predicted *HoxB1a* and *HoxB3a* 3′UTR and ORF target sites as mediators of *miR-10* repression.

### Response of endogenous Hox genes to miR-10 gain and loss of function

**Figure 3:**
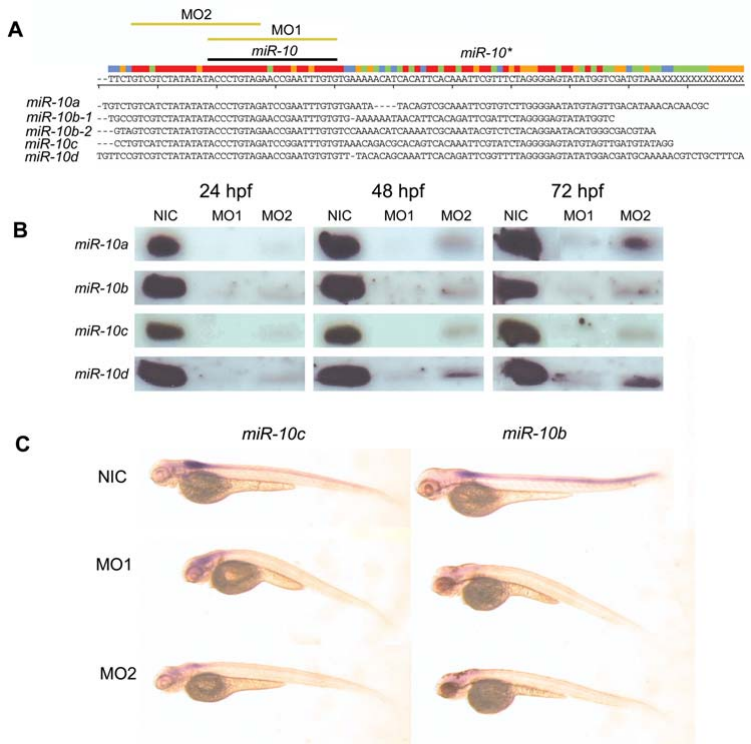
Morpholino knockdown of *miR-10.* A) ClustalW alignment of the 5 Zebrafish *miR-10* precursor sequences. Indicated are the positions of the mature microRNA, the hairloop and the miR-10* (antisense pairing sequence in the hairpin). The target sequences for both morpholino reagent 1 and 2 are indicated with yellow bars (MO1 and MO2). B) Northern blot for all 4 different *miR-10* isoforms in morpholino injected embryos at 24, 48 and 72 hpf. There is an absence or very strong downregulation of the mature microRNA in the morpholino injected samples. C) LNA *in situ* hybridization for *miR-10b* and *miR-10c* in 72 hpf *miR-10* morphants. The endogenous expression of *miR-10b* and *miR-10c* is no longer detected.

To investigate the role of *miR-10* in the regulation of endogenous *Hox* genes, we performed *miR-10* gain and loss of function experiments. For gain of function, the injection of a siRNA into the zygote is an effective way to overexpress microRNAs [35]. Loss of function can be achieved via the injection of antisense morpholinos. Processing and production of a mature miRNA are effectively blocked by a morpholino directed against a microRNA precursor [22]. As morpholinos allow mismatches with the target sequence, it is possible to target several miRNA isoforms using fewer morpholino sequences. ClustalW alignment of the 5 *miR-10* paralogue precursor sequences reveals a region of extended conservation in the stem loop 5′ to the mature microRNA (figure 3A), which allows the design of two morpholino reagents with only minor overlap to control against off-target effects. Morpholino reagent 1 (MO1) consists of a mix of two morpholinos directed against the *miR-10a* and *miR-10b* mature sequences. Morpholino reagent 2 (MO2) is a single morpholino directed against the upstream conserved sequence (figure 3A). Either morpholino has maximally one nucleotide mismatch with any of the 5 *miR-10* paralogues. Injection of either MO1 or MO2 leads to absence or very strong reduction of the signal for each of the 4 *miR-10* isoforms in northern blots (figure 3B), showing that their processing is efficiently inhibited. For MO2 we observe a slight recovery of the signal at 48 and 72 hpf (hours post fertilization), but overall, injection results in a very strong decrease of mature *miR-10* levels. *In situ* hybridization with *miR-10* LNA probes also shows no signal in morpholino injected embryos (figure 3C).

Besides interfering with translational processes, targeting by microRNAs leads to reduced transcript stability and decreases the amounts of transcript present [20], [36]. RNA levels can therefore function as read out of a transcript/microRNA interaction [20].

Embryos were injected with either *miR-10* siRNA or *miR-10* morpholino and then analyzed at 24 hpf for the expression of the target genes *HoxB1a* and *HoxB3a*, and of *HoxB2a*, *HoxB4a* and *HoxB5a*, genes that are predicted not to be targets.

**Figure 4:**
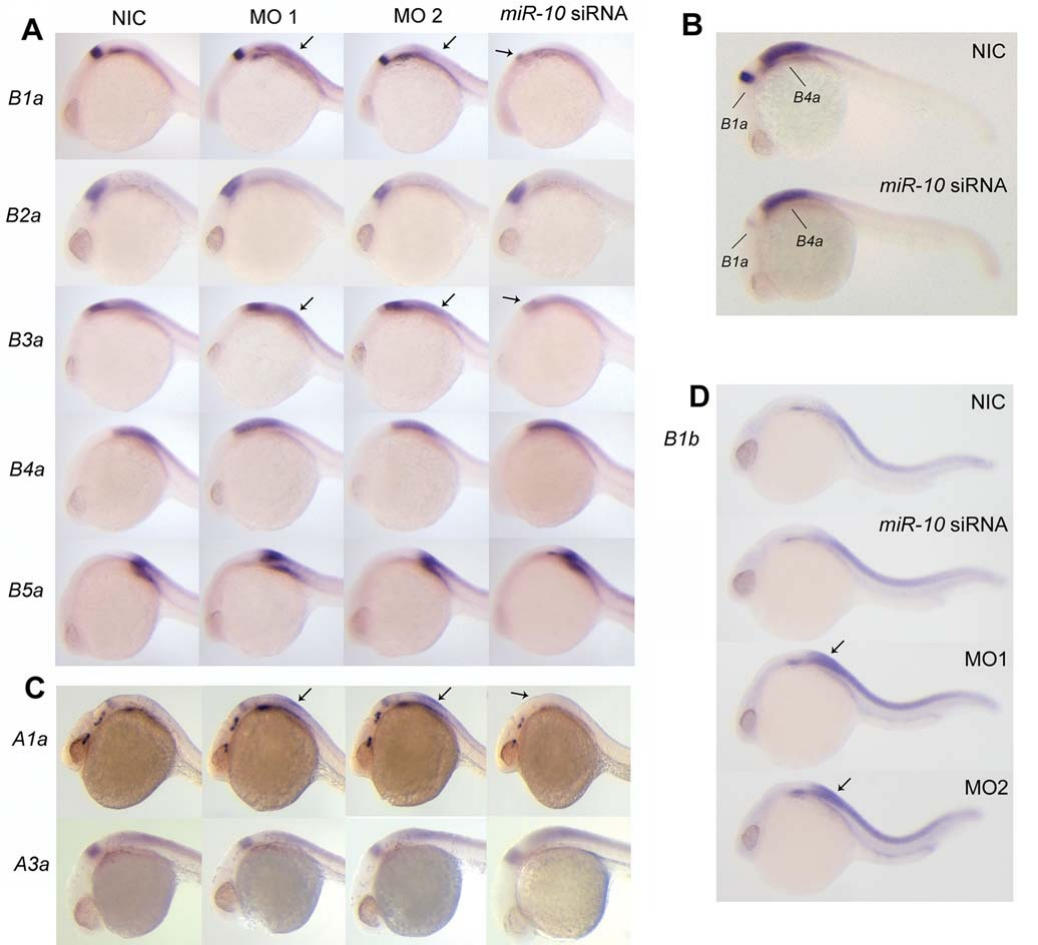
Effect of *miR-10* knockdown and overexpression on endogenous *Hox* target transcripts. A) Whole mount *in situ* hybridization with probes for *hoxB1a*, *B2a*, *B3a*, *B4a* and *B5a* on 24hpf embryos injected with morpholino reagent 1 or 2 (MO1 or MO2), *miR-10* siRNA or non injected controls (NIC). To allow quantitative detection, embryos hybridized with the same probe were stained equally long and staining was continuously monitored and stopped before reaching signal saturation. *HoxB1a* and *HoxB3a* respond to both gain and loss of function (arrows) of *miR-10* with a decrease and increase in expression levels respectively. *HoxB2a*, *HoxB4a* and *HoxB5a* are unresponsive to *miR-10* overexpression or knockdown. B) Double whole mount *in situ* hybridization on 24 hr embryos using probes for *hoxB1a* and *hoxB4a* showing the different responses of the genes. Embryos were stained equally long till adequate staining was obtained for the *hoxB4a* probe. C) *In situ* hybridization with *HoxA1a and HoxA3a* on 24 hpf embryos injected with MO1, MO2 or *miR-10* siRNA. *HoxA1a* responds to both overexpression and knockdown. The transcript level of *HoxA3a* does not respond to either overexpression or knockdown. D) *In situ* hybridization with *HoxB1b* on 24 hpf embryos morphant and overexpression embryos. There is strong upregulation of *HoxB1b* expression in the morphants but no downregulation is observed in the *miR-10* siRNA injected embryos.

Overexpression of *miR-10* leads to downregulation of *HoxB1a* and *HoxB3a* in their strong anterior hindbrain expression domains but does not influence the expression of the other *Hox* genes (figure 4A marked *miR-10* siRNA). *In situ* hybridization with the ‘sensor part’ of *HoxB3a* shows that this region responds identically to overexpression of *miR-10* (figure S1B). In the morpholino injected embryos, increased *HoxB1a* expression is observed in the hindbrain/spinal cord transition (figure 4A). *HoxB3a* expression shows a similar posterior upregulation in morphant embryos, although to a lesser extent (figure 4A). The other *Hox* genes, which are also not affected by the overexpression of *miR-10* siRNA, show no change in expression levels in morphant embryos. Relative quantitativity of the method was assessed by control double *in situ* hybridization using *HoxB1a* and *HoxB4a*, which shows that it is possible to visualize the different responses of these genes (figure 4B).

Additional *Hox-1* and *Hox-3* paralogue members are located in the *HoxAa*, *HoxBb*, *HoxCa* and *HoxDa* clusters. In *HoxA3a*, one putative *miR-10* target site is present in the *HoxA3a* 3′ UTR (777 nt downstream of the ORF), in *HoxB1b* one putative target site is located in the 3′ UTR (125 nt downstream of the ORF) and in *HoxA1a*, a seed sequence is located 5472 nt downstream of its ORF.

No seed sequences are associated with the *HoxC1a*, *HoxC3a* or *HoxD3a* coding regions or 3′ UTRs. We also determined the responses of *HoxA3a*, *HoxA1a* and *HoxB1b* to overexpression and knockdown of *miR-10*. Surprisingly, both *HoxA1a* and *HoxB1b* are strongly upregulated in the spinal cord in *miR-10* morphant embryos (figure 4C, E), suggesting either direct de-repression by *miR-10* or activation by the now de-repressed *HoxB1a* gene. In addition *HoxA1a* also responds strongly to overexpression of the *miR-10* siRNA. We don't observe any changes in the expression of *HoxA3a* resulting from overexpression or knockdown of *miR-10* (figure 4C).

### HoxB1a upregulation by retinoic acid is elevated in miR-10 morphants

**Figure 5:**
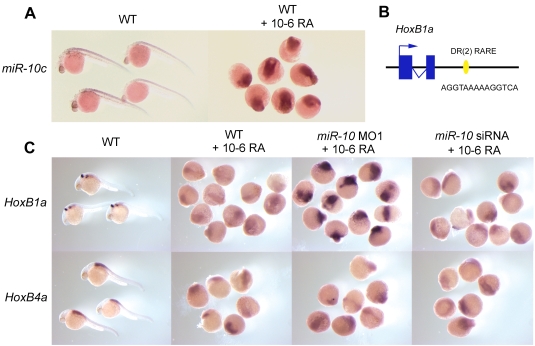
Retinoid induction of *miR-10c* and upregulation of *HoxB1a* in *miR-10* morphants. A) LNA *in situ* hybridization for *miR-10c* in wildtype (WT) and 10^−6^M retinoic acid (RA) treated embryos. RA treatement results in *miR-10c* upregulation. B) Presence of a DR[2] type retinoic acid response element (RARE) 1kb 3′ of the Zebrafish *HoxB1a* gene. This sequence is conserved in the mouse in which it has been shown to mediate the neural response of *HoxB1* to RA [38] C) Different response of *HoxB1a* to RA stimulation in wildtype or *miR-10* morphant embryos. *HoxB1a* is strongly upregulated in *miR-10* morphants. Injection with the *miR-10* siRNA has no effect. *HoxB4a* responds similar to all conditions.

Anterior *Hox* genes are regulated in early developing central nervous system by retinoic acid (RA) [27] and possess cis-acting retinoid response elements [37]. The co-expression and implied co-regulation of *miR-10c* with *HoxB4a* suggest that it could be regulated in the same way. *In situ* hybridization shows that treatment with 10^−6^ M RA leads to upregulation of *miR-10c* (figure 5A), in a manner similar to that of *HoxB4a*, showing that transcription of *miR-10c* is indeed activated by RA.

In mouse, *HoxB1* possesses several retinoid response elements and retinoids play a role in the establishment of the endogenous neural expression pattern [38], [39], [40], [41]. The 3′ DR[2] type retinoid response element, which has been shown to regulate neural RA responsiveness of the mouse *HoxB1* gene [38], is conserved in the Zebrafish *HoxB1a* gene (figure 5B). In RA stimulated Zebrafish embryos, *HoxB1a* expression is indeed no longer restricted to a single strong domain (figure 5C) and this gene is expressed much more extensively throughout the embryo, suggesting activation. There is, however, a decrease in expression level, which is remarkable in the light of the presumed activating activity of RA. We investigated whether the co-activation of *miR-10* plays a role in this. When *miR-10* knockdown embryos are stimulated with 10^−6^ RA, an increased upregulation of *HoxB1a* but not *HoxB4a* is observed (figure 5C) compared to wildtype treated embryos. Apparently RA simultaneously activates expression of both *HoxB1a* and *miR-10* and *miR-10* modulates the downstream response to retinoid signaling.

### Overexpression of miR-10 induces phenotypes associated with loss of HoxB1a and HoxB3a but not HoxB1b

**Figure 6:**
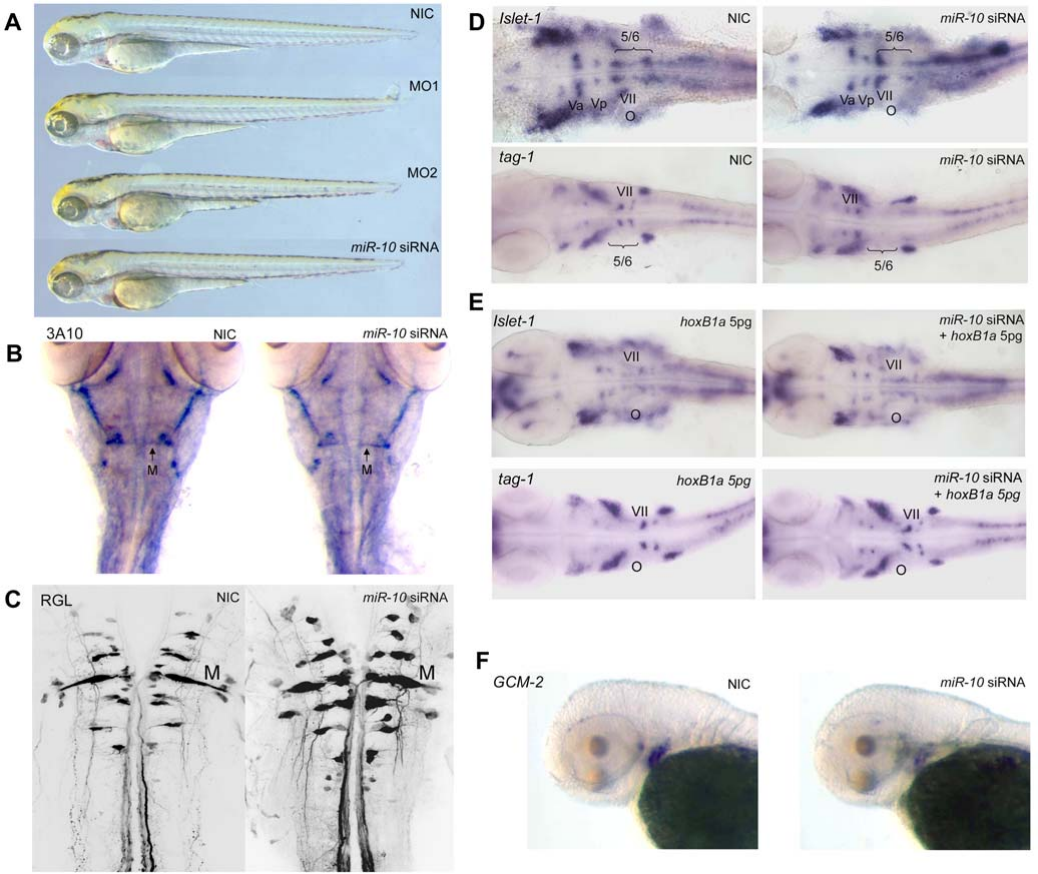
Overexpression of *miR-10* induces *HoxB1a* and *HoxB3a* loss of function phenotypes. A) Wildtype (WT), *miR-10* morphant (MO1, MO2) and *miR-10* siRNA overexpression embryos at 72 hpf show no apparent developmental differences. B) Mauthner neuron development as visualized by 3A10 neurofilament immunostaining in 72 hpf embryos shows no differences between *miR-10* siRNA injected embryos and controls. C) Confocal images of reticulospinal hindbrain neurons in retrograde labeled, 5 day old embryos. Wildtype and *miR-10* siRNA injected embryos are similar. D) *Islet-1* and *tag-1 in situ* hybridization on 30 hpf wildtype and *miR-10* siRNA injected embryos. Flatmounts of head regions are shown. In wildtype embryos the VIIth cranial nerve migrates into rhombomere 5/6 at the level of the otic vesicle. In *miR-10* siRNA injected embryos the VIIth nerve does no longer migrate out of rhombomere 4. E) Co-injection of 5pg *HoxB1a* RNA rescues the *miR-10* siRNA induced migration defect of the VIIth cranial nerve as shown by *islet-1* and *tag-1 in situ* hybridization. F) *Gcm-2* expression is downregulated in *miR-10* siRNA injected embryos, which is consistent with repression of *HoxB3a*.

The phenotypes of morphant and overexpression embryos are remarkably normal during the first 5 days of development and they have no apparent defects (figure 6A, 72 hpf embryos shown). However, as *miR-10* appears to target *HoxB1a* and *HoxB3a* and possibly other *1* and *3* paralogue genes, the phenotype of *miR-10* overexpression is expected to combine at least the loss of function phenotypes for these genes.

In Zebrafish, morpholino studies have shown that *HoxB1a* is required for the correct patterning of rhombomere 4 together with *HoxB1b* [42]. Single knockdown of *HoxB1a* results in a failure of the branchiomotor neurons of the VIIth cranial nerve to migrate out of rhombomere 4. In the double knockdown of *HoxB1a* and *HoxB1b,* there is additional absence of the rhombomere 4 primary Mauthner neurons.

Morpholino knockdown of *HoxB3a* and *HoxA3a* has been shown to result in downregulation of the *gcm-2* gene in the branchial arches in Zebrafish [43].

Analysis of the *miR-10* overexpression phenotype by immunolabeling with the primary neuron specific 3A10 antibody shows that the Mauthner neurons are still present (figure 6B). Retrograde labeling in 5 days old embryos also reveals a normal pattern of reticulospinal neurons projecting from the hindbrain into the spinal cord (figure 6C).

Hindbrain branchiomotorneurons were visualized in *in situ* hybridization with *islet-1* and *tag-1. Islet-1* stains the bodies of the Vth, VIIth, IXth and Xth nerves and *tag-1* is specifically expressed in the migrating VIIth nerve.

*In situ* hybridization on 30 hpf *miR-10* injected embryos shows that branchiomotor neurons of the VIIth nerve no longer migrate into rhombomere 5 and 6 but remain in rhombomere 4 (figure 6D). The pattern of the Vth, IXth and Xth branchiomotor nerves as visualized by *islet-1* appears normal. To show that the VIIth nerve defect is directly due to targeting of *HoxB1a* by *miR-10*, we rescued the *miR-10* overexpression by co-injecting 5pg *HoxB1a* RNA from a construct that does not contain any of the target sites.

Injection of 5 pg *HoxB1a* alone does not induce any phenotype. Co-injection with *miR-10* siRNA restores migration of the VIIth nerve as show by both *islet-1* and *tag-1 in situ* hybridization (figure 6E).

To show targeting of *HoxB3a*, 72 hpf embryos were analyzed for the expression of *gcm-2.* In *miR-10* overexpression embryos we observe downregulation of *gcm-2* (figure 6F) in the branchial arch region as would be expected for embryos with impaired *HoxA3a* and/or *HoxB3a* expression [43].These analyses show that *miR-10* is able to induce specific phenotypes associated with the loss of function of *HoxB1a* and *HoxB3a*/*HoxA3a* genes but not of *HoxB1b*. Analysis of the same genes in *miR-10* morphant embryos shows patterns similar to wildtype embryos (figure S3).

### MiR-10 acts synergistically with HoxB4

**Figure 7:**
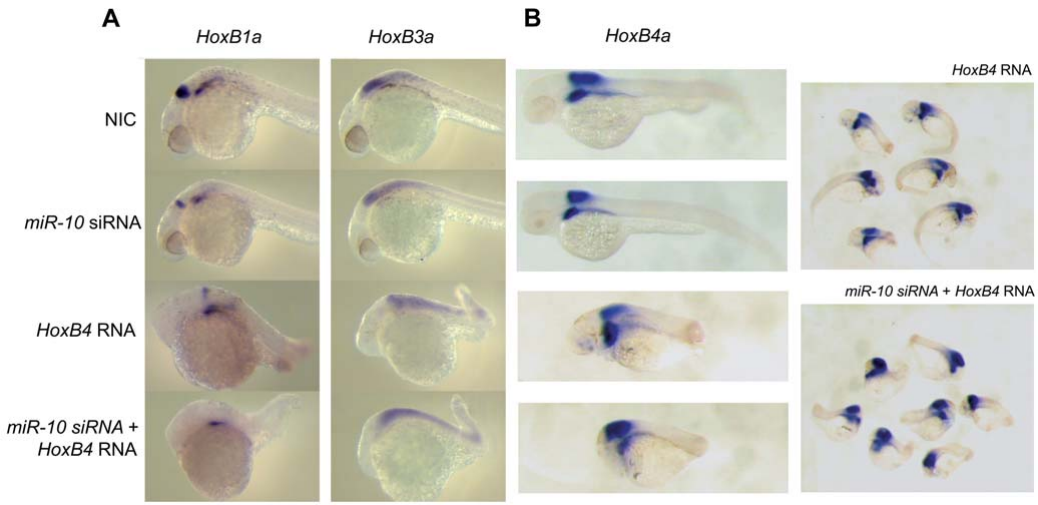
* MiR-10* acts in synergy with *HoxB4.* A) Embryos injected with *HoxB4*, *miR-10* siRNA and *HoxB4*+*miR-10* siRNA analyzed for the expression of *HoxB1a* and *HoxB3a* at 24 hpf. Injection of 150pg *HoxB4* leads to downregulation of *HoxB1a* and of downregulation of the hindbrain domain of *HoxB3a*. The rhombomere 4 expression domain of *HoxB1a* and the rhombomere 5/6 expression domain of *HoxB3a* are still discernable though. When 150pg *HoxB4* is expressed together with *miR-10* siRNA the expression domain of *HoxB1a* disappears and no clear rhombomere 5/6 stripe of *HoxB3a* expression can be detected. B) Embryos injected with *HoxB4*, *miR-10* siRNA and *HoxB4*+*miR-10* siRNA analyzed for the expression of endogenous *HoxB4a* at 48 hpf. The combination of *HoxB4* together with the *miR-10* siRNA induces a stronger phenotype with more severe anterior and posterior truncations than injection with *HoxB4* alone. On the right groups of embryos injected with *HoxB4* or the combination of *HoxB4* and *miR-10* siRNA are shown.

In *Xenopus laevis,* overexpression of *HoxB4* has been reported to repress the expression of *HoxB1* and *HoxB3* in neuralized animal caps [44]. Considering that these are the same genes that are targeted by *miR-10* and that there exists a close association between the microRNA gene and the *HoxB4* open reading frame, this could indicate a synergistic action between *miR-10* and *HoxB4*. To test this hypothesis we overexpressed *HoxB4* and *miR-10* individually and combined. Embryos were injected with 150pg *hoxB4* RNA, *miR-10* siRNA or a combination of the two and analyzed for *HoxB1a* and *HoxB3a* expression. Injection of 150 pg *Xenopus laevis HoxB4* RNA strongly represses the hindbrain rhombomere 4 expression of *HoxB1a* and rhombomere 5/6 expression of *HoxB3a* (figure 7A).

However, for *HoxB1a* there is still a weak r4 domain detectable and for *HoxB3a* a discrete r5/6 stripe of expression is present. When coexpressed with *miR-10*, we observe a complete disappearance of the rhombomere 4 *HoxB1a* expression stripe (figure 7A). For *HoxB3a*, there still is expression in the hindbrain but a discrete r5/6 domain is no longer discernable (figure 7A). In addition to this, we observe a stronger phenotype at 48 hpf, with more severe anterior and posterior truncations in embryos injected with the combination of *HoxB4* RNA and *miR-10* siRNA (figure 7B). *In situ* hybridization for the endogenous *HoxB4a* shows that in the *HoxB4*+*miR-10* siRNA co-injected embryos, the parts anterior and posterior to the endogenous *HoxB4a* domain are reduced more strongly than in embryos injected with *HoxB4a* only. These experiments indicate that *miR-10* synergizes with *HoxB4* in the repression of *HoxB1a* and *HoxB3a* and also attains a greater posteriorizing activity in the presence of *miR-10*.

### Evolutionary conservation of the target sites in the HoxB cluster

**Figure 8:**
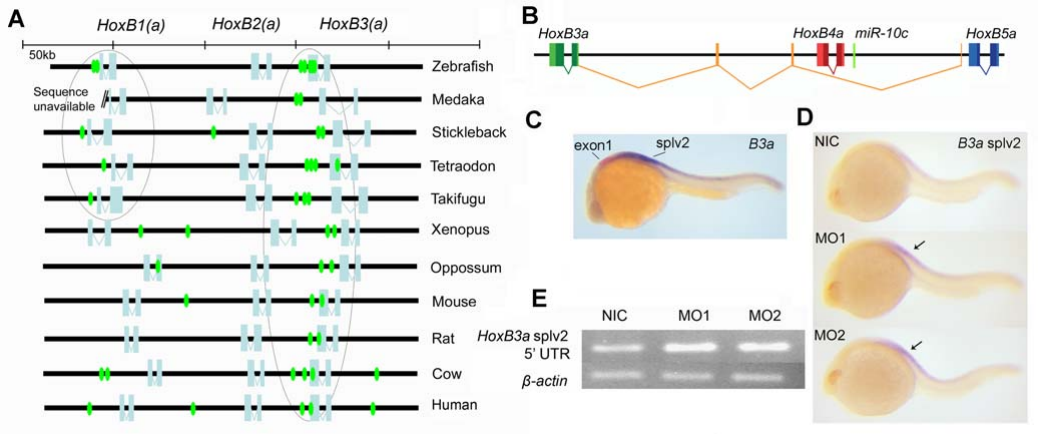
Evolutionary conservation of *miR-10* targetsites and autoregulation of *miR-10c.* A) Putative *miR-10* target sites are indicated by seed sequences in the sense strand of the anterior vertebrate *HoxB(a)* clusters. Seed sequences are shown in green, open reading frames are indicated in light blue. Note conserved association of target sites with the *HoxB3(a)* ORF and conserved presence of a putative target site in *Teleost HoxB1a*. B) The *HoxB3a* splv2 polycistronic transcript includes one exon between *HoxB4a* and *HoxB5a*, two exons between *HoxB4a* and *HoxB3a* and the main *HoxB3a* coding sequence. The primary transcript for this isoforms includes *miR-10c*. The 5′ UTR sequence is shown in orange, this sequence corresponds to the probe used in C and D to specifically detect this splice isoforms. C) Comparison of the *HoxB3a* exon1 expression (red) and the expression of *HoxB3a* splv2 (purple). *HoxB3a* splv2 is expressed posterior to the main rhombomere 5/6 expression domain of *HoxB3a* as reported previously [46]. The staining reaction for *HoxB3a* splv2 was developed for much longer than the reaction for the *HoxB3a* exon1 probe and the *HoxB3a* splv2 is presumably expressed at a much lower level. D) *In situ* hybridization with *HoxB3a* splv2. Expression is upregulated in *miR-10* morphant embryos (arrows). E) Semi quantitative RT-PCR for the *HoxB3a* splv2 5′ UTR, *ß-actin* is used as loading control. *HoxB3a* is upregulated in *miR-10* morphant embryos. *HoxB3a* splv2: 31 cycles, *ß-actin*: 22 cycles.

Evolutionary conservation of sequence information is considered a good indicator of functionality and is used in microRNA target prediction programs [34] to assign confidence levels. We searched the anterior part of the *HoxB(a)* clusters in Medaka, Three spined stickleback, *Tetraodon*, *Takifugu*, *Xenopus*, Oppossum, Mouse, Rat, Cow and Human for the presence of putative *miR-10* target sites (seed nucleotide 1-7). In figure 8A, the anterior parts of the *HoxB* and *HoxBa* cluster homologues are shown with indication of the identified seed sequences. The conservation of the *miR-10* target sites in the 3′UTR and coding regions of *HoxB3(a)* genes is clear; all species investigated have at least 2 target sites associated with the ORF or 3′ UTR region. The sites in *HoxB1(a)* show a weaker conservation profile and are most prominently present in Zebrafish. All of the *Teleost*s for which sequence information could be found (note that the available *HoxBa* Medaka contig stops 300 nt downstream of *HoxB1*) posses a candidate *miR-10* target site in the 3′ UTR of their *HoxB1a* gene. This further adds to the implied relevance of the repression of *HoxB1a* and *HoxB3a* by *miR-10*.

### A polycistronic transcript including both HoxB3a and miR-10c is targeted by miR-10

A transcript, *HoxB3a* splv2 [45], has been described, which starts 3′ of *HoxB5a*, has two exons originating 3′of *HoxB4a* and includes the main *HoxB3a* open reading frame (figure 8B). The primary unspliced form of this long transcript thus includes both *HoxB3a* and the *miR-10c* microRNA.

*In situ* hybridization with a 5′ UTR probe shows that expression of this transcript obeys the rules of colinearity and that its rostral expression boundary thus corresponds to the position of its transcriptional start site (i.e. expression similar to that of *HoxB5a*) (figure 8C) and [46]. This transcript is thus expressed more posteriorly than the main *HoxB3a* expression domain and completely within the domain of the *miR-10c* microRNA, a feature also expected from the presence of *miR-10c* on the *HoxB3a* splv2 primary transcript. This transcript includes the full *HoxB3a* open reading frame together with the 3 *miR-10* target sites. Morpholino knockdown of m*iR-10* leads to upregulation of this transcript in both *in situ* hybridization and RT-PCR (figure 8D, E), confirming that it is indeed targeted by *miR-10* in vivo. In this case the *miR-10c* microRNA apparently acts on parts of its own primary transcript and is therefore autoregulatory.

## Discussion

**Figure 9:**
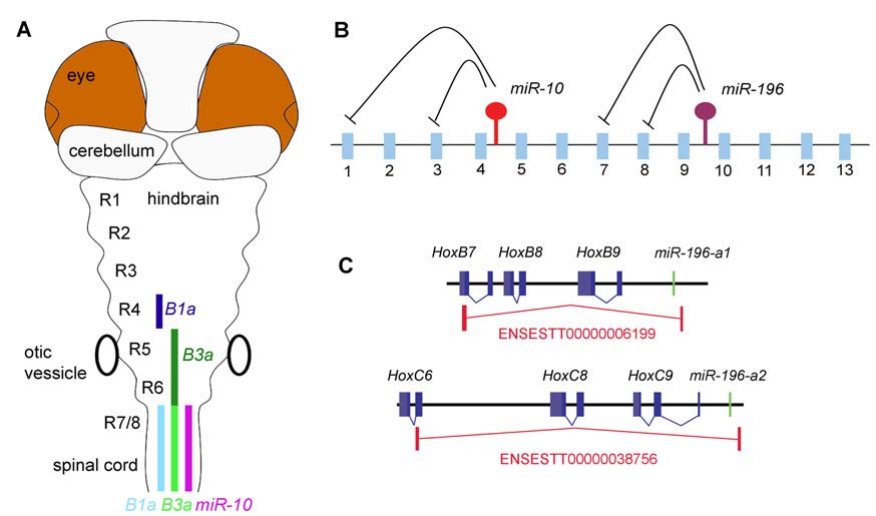
Post-transcriptional regulatory interactions within the hox clusters. A) Schematic representation of *miR-10* and target gene expression in the Zebrafish hindbrain. *MiR-10* is expressed posterior from the rhombomere 6/7 boundary. The target genes *HoxB1a* and *HoxB3a* are expressed in a strong domain (dark colour) anterior in the anterior hindbrain and in a weaker domain (light colour) in the area where they overlap with *miR-10*. *HoxB1a* shows a gap in expression in r5 and 6, possibly due to stronger transcriptional repression. B) Schematic representation of the post-transcriptional relations within the hox clusters. *MiR-196* is known to represses *HoxB8*, *HoxC8*, *HoxD8* and *HoxA7* and we have identified *HoxB1a* and *HoxB3a* as targets for *miR-10*. The emerging view is that the microRNAs in the hox clusters target more anterior genes in their close proximity. C) Polycistronic transcripts identified from the EST database show inclusion of *miR-196* paralogues and *HoxB8* and *HoxC8* target genes on the same primary transcripts.

*MiR-10* is expressed in the hindbrain and spinal cord posterior to the rhombomere 6/7 boundary and occupies an axial domain similar to those of *Hox-4* paralogue genes. We reveal an interaction between *miR-10* and the anterior *HoxB1a* and *HoxB3a* genes. These *Hox* genes have strong anterior expression domains in the hindbrain and are expressed at a low level in the spinal cord where their expression overlaps with *miR-10* expression (figure 9A). The upregulation of the target genes in the morphant embryos shows that the target genes are indeed repressed by *miR-10* within this posterior domain.

Overexpression of *miR-10* indeed induces the phenotypes associated with the loss of *HoxB1a* and *HoxB3a*. The very specific phenotype induced in the overexpression experiments is striking. The embryos are virtually indistinguishable from wildtypes, apart from the VIIth nerve defect and altered *gcm-2* expression. In the target prediction section of miRBase, where the output of the Miranda algorithm (http://microrna.sanger.ac.uk/) is listed, there are however 1969 predicted target genes for Zebrafish *miR-10*. This high number of predicted target genes seems incompatible with the very specific phenotypic defects observed in the *miR-10* overexpression embryos and strongly suggest that, at least for *miR-10*, there is a high component of false positives in the outcome of target prediction algorithms.

We observe that the *HoxB4* overexpression phenotype is significantly enhanced by *miR-10*. The close genomic association of these two genes and their synergistic activity could indicate that these genes are part of the same genetic unit. The system of *Hox* regulation is characterized by the phenomenon of posterior prevalence, meaning that there is a hierarchy in the functioning of the *Hox* genes, such that posterior genes are always dominant in the determination of a regional phenotype over coexpressed anterior genes [2]. In the overexpression experiments it looks as if miR*-10* facilitates a full posteriorizing activity of *HoxB4*. This synergistic action between *miR-10* and *HoxB4* suggests that post-transcriptional gene regulation by microRNAs plays a role in posterior prevalence.

We find that *HoxB1a* and *HoxB3a* are targeted by *miR-10*. Other *Hox-1* and *Hox-3* paralogues genes are present in different Zebrafish *Hox* clusters but are not targeted. On the basis of our experiments and the presence of putative target sites, only *HoxA1a*, *HoxB1a*, *HoxA3a* and *HoxB3a* genes are candidate *miR-10* targets. Whether they are true targets remains to be seen; *HoxA1a*, despite not being near to a clear target site, responds strongly to the loss and gain of *miR-10*. *HoxB1b* possesses a candidate target site and is upregulated in morphant embryos. What however argues strongly against the targeting of *HoxB1b* is that the overexpression of *miR-10* does not induce the same phenotypic changes as observed in the double *HoxB1a*/*HoxB1b* knockdown [42]. As there is extensive crossregulation between *Hox* paralogues it is also possible that the effects observed are a direct result of the derepression of *HoxB1a*. *HoxA3a* also possesses one candidate target site but seems unaffected by gain and loss of *miR-10*. MicroRNAs affect target genes both by inhibition of translation and by degradation of messengerRNA [20], [36]. To what extent these processes are coupled and whether translational inhibition is always accompanied by an increase in messenger RNA decay is not yet known. It is thus theoretically possible that the effect of *HoxA3a* repression will only be noticeable at the protein level.

There could be several reasons why *miR-10* targets particular *Hox-1* and *Hox-3* genes and not others. One explanation would be that there is a high degree of subfunctionalization within these paralogue groups, as was nicely illustrated for the Zebrafish *Hox-1* genes [42]. This could create needs for post-transcriptional silencing that differ from one paralogue member to another.

The inhibition of *miR-10* leads to posterior upregulation of the targeted genes. Since microRNAs cause downregulation of their target messenger RNAs, it has been a frequently debated issue whether low or absent levels of target gene expression within the microRNA domain reflect different domains of transcription or whether they are a direct consequence of the downregulation by the microRNA [e.g. 47]. Inhibition of *miR-10* leads to posterior target gene upregulation in case of *HoxB1a*, *HoxA1a* and *HoxB3a* but certainly not to the same high level found in their dominant anterior expression domains. It thus appears that restriction to the dominant expression domain occurs primarily at the transcriptional level and that it is within a posterior domain with an already low level of transcription that silencing by the microRNA occurs. This observation is consistent with the identification of rhombomere specific transcriptional *Hox* enhancers in mouse [e.g. 48], [49]. In case of the microRNA target interactions described in this study, it seems that both different transcriptional domains and a direct repression by the microRNA shape the mRNA expression domains in the embryo. The effects at the transcript level likely reflect an on/off situation at the protein level where all translation is silenced although there is still a significant amount of messengerRNA detectable.

### Why are there post-transcriptional gene regulatory interactions within the Hox clusters?

In general it is poorly understood why functional domains of genes are sometimes restricted post-transcriptionally instead of by transcriptional silencing. The emerging view for the microRNAs in the *Hox* clusters is that they target coding *Hox* genes (figure 9B) that can even be located within the same clusters. These microRNAs thus seem to be involved in post-transcriptional gene regulatory interactions with genes that are located in their very close vicinity. The short genomic distances between *miR-196* and *miR-10* and their targets are remarkable; *miR-10*c is ∼25 kb from the target sites in *HoxB3a* and ∼48 kb from those in *HoxB1a* and (in mammals) a *miR-196* paralogue is located at ∼18 kb from *HoxB8* and *HoxC8* and at ∼14 kb from *HoxA7*.

In this light, the presence of *miR-10c* and its target *HoxB3a* on a single primary transcript is also interesting. The *miR-10c*/*HoxB3a* polycistronic transcript includes both the microRNA and a target gene. In this case, the microRNA thus acts in an autoregulatory fashion on parts of its original precursor. The *HoxB3a* splv2 transcript itself appears to be expressed exclusively within the expression domain of the microRNA and is thus never expected to be translated into a functional *HoxB3a* protein. Similar transcripts are present in the EST database for *miR-196a-1*/*HoxB8* and *miR-196a-2*/*HoxC8* (figure 9C). The inclusion of microRNAs and target genes on the same transcription unit is counterintuitive in the sense that one wonders why the target genes are not simply omitted from the transcript as they are silenced by the accompanying microRNA anyway.

A possible explanation for the presence of target gene/microRNA combinations within single transcription units and for the targeting of nearby *Hox* genes by both *miR-10* and *miR-196*, may lie in the complexity of the *Hox* regulatory mechanisms which involve multiple global and local transcriptional elements. The high selective pressure to maintain the clustered genomic organization of vertebrate *Hox* genes probably results from the presence of global enhancers located outside of the clusters and from dependence on sharing of local enhancers [50]. As a result, the *Hox* clusters consist of closely spaced transcription units and enhancer regions. The high density of transcription units could easily cause them to interfere with one another and make the system prone to inappropriate enhancer sharing, resulting in ectopic expression. The extensive amount of ‘strange’ polycistronic and antisense transcripts being produced from the *Hox* clusters [51] could result from this. These transcripts do not necessarily have any function but could represent inherent transcriptional ‘noise’. It is interesting that it is specifically the nearest genes that are silenced post- transcriptionally as these are the ones most likely to be influenced by the same enhancers as the microRNA genes. The posterior expression domains of these anterior *Hox* genes could well be a consequence imposed on the transcriptional process by the clustered nature of the genes and they do not necessarily serve any function. We suggest that an inability to separate the transcriptional controls of several *Hox* genes is the selective force driving the post-transcriptional gene silencing relationships within the *Hox* clusters.

In vertebrates, *Hox* genes have stayed clustered throughout evolution. However, in the sister group of tunicates (*Ciona* and *Oikopleura*), the *Hox* clusters have broken up and are present in separate regions of the genome [52], [53]. It is interesting to note that the *miR-10* microRNA has been lost from the tunicates [17], [54]. This observation provides a possible phylogenetic link between post-transcriptional gene silencing and gene clustering.

It would be interesting to see whether it is possible to extrapolate these observations to other microRNA/(predicted) target pairs and see if similar constraints can be identified that possibly account for the involvement of post-transcriptional gene regulation.

## Materials and Methods

### Zebrafish husbandry and embryo culturing

An AB×TL strain of Zebrafish was used for all experiments; housing and embryo collection was according to standard procedures; embryos were cultured at 28°C.

### RT-PCR

Whole embryo RNA was isolated using Tri-pure (Roche #1667165) and reverse transcribed with MuMlv Reverse transcriptase (Promega) using oligo-dT N = 18.

Primer sequences;

1. *miR-10c* up (AGCTGGCTTTCTCAATACC)
2. *miR-10c* dow n (TACATACTCCCCTAGATACGAA)
3. *HoxB4a* exon1 up (ATGGCCATGAGTTCCTATTTG)
4. *HoxB4a* exon1 down (TTGGTTCACCCCCTGAATAG)
5. *HoxB4a* exon1 5′down (TTGTGGGTAGAACGTGACCTC)
6. *HoxB3a* splv2 5′ UTR up (CAGTGCCAGTGTCTAGTCAG)
7. *HoxB3a* splv2 5′UTR down (GTAATACGACTCACTATAGGCTCTTTCCAATGGCCTCTTGG)
8. *β-Actin* up (CGAGCAGGAGATGGGAACC)
9. *β-Actin* down (CAACGGAAACGCTCATTGC)

DNA oligos were obtained from Biolegio, Malden, The Netherlands.

### Micro-injection

Embryos were injected with 1 or 2 nl at the zygote stage; RNAse free phenol red was added as tracer to injection mixtures prior to injections.

Morpholinos were obtained from genetools, OR, USA; *miR-10* morpholino reagent 1 corresponds to a mix of *miR-10a* (CACAAATTCGGATCTACAGGGTA) and *miR-10b* (CACAAATTCGGTTCTACAGGGTA) antisense morpholino, the sequence of *miR-10* morpholino reagent 2 is (TCTACAGGGTATATATAGACGAC).

RNA oligos were obtained from Biolegio, Malden, The Netherlands.

The *miR-10* siRNA sense strand corresponds to a mix of *miR-10a* (UACCCUGUAGAUCCGAAUUUGUGUG) and *miR-10b* (UACCCUGUAGAACCGAAUUUGUGUG), sequence of the antisense strand is (CACAAAUUCGGAUCUACAGGGGCAU). Note that the antisense sequence has mismatches with the *miR-10* sense strand at its 3′ end resulting in the specific incorporation of the sense *miR-10* strand in the microRNA silencing complex. Oligos were annealed to siRNAs in by gradually cooling from 98°C to 20°C. in buffer in 500ml H_2_O beaker glass; 30 ul 50 µM of each oligo, 15 µl annealing buffer (50 mM Tris, pH 7.8, 100 mM NaCl RNAse free) in 75 µl, final concentration of siRNA is 20 µM.

RNA for injection was transcribed using Ambion Sp6 message machine kit (# 1340) and purified using an RNA easy column (Qiagen), from the CS2+ plasmids; CS2+*HoxB1a* sensor wt, CS2+*HoxB1a* sensor mut, CS2+*HoxB3a* sensor wt, CS2+ *HoxB3a* sensor mut, CS2+Dre-*HoxB3a* ORF, CS2+Xl-*HoxB4*-Myc, CS2+*E-YFP*, CS2+*E-CFP*.

### In situ Hybridization

*In situ* hybridization was performed according to standard procedures and Kloosterman et al. [32]. Hybridization temperatures were 65°C for normal probes and 56°C for LNA probes. In double *in situ* hybridization with a LNA probe 56°C was used. In double *in situ* hybridization DIG and fluorescein labeled probes were used. Embryos were stained using BM-Purple (Roche #11442674001) and Fast Red (Roche #11496549001). Probes were synthesized using T7 and Sp6 polymerase (Promega) in the presence of labeled nucleotides (RNA DIG or fluorescein labeling mix, Roche #11277073910 and #10805221) from pGEM-TE plasmids containing: *HoxB1a*, *HoxB3a, HoxB4a and HoxB5a* exon 1 coding sequence, *HoxB2a* exon 2-3′UTR, *HoxB1b* exon1-2 coding sequence; *Hox*B3a splv2 was synthesized from PCR product from a partial cDNA cloned in pGEM-TE.

LNA probes were obtained from Exiqon, Denmark and sequences are:

1. *miR-10a* (CACAAATTCGGATCTACAGGGTA),
2. *miR-10b* (ACAAATTCGGTTCTACAGGGTA),
3. *miR-10c* (CACAAATCCGGATCTACAGGGTA),
4. *miR-10d* (ACACATTCGGTTCTACAGGGTA ).

Probes were labeled using the DIG labeling kit (Roche #03353575910) and purified before use over a microspin G-25 column (Amersham #27-5325-01) Our step by step *in situ* hybrdization protocol is available on request.

### Nothern Blot

Northern Blot was performed essentially according to Kloosterman et al. [55]. Total RNA was extracted using Tri-Pure (Roche #1667165). 3 µg RNA was separated on a 15% denaturing PAGE gel using a Biorad minigel system and subsequently blotted using a semidry blotter (175 mA constant, 10–20V for 25–30 min.) to a postitively charged nylon membrane (Roche #1417240). Membranes were pre-hybridized at 60°C for 1 hr in hybridization buffer (0.36 M Na_2_HPO_4_, 0.14M NaH_2_PO_4_, 1 mM EDTA, 7%SDS, 0.1 mg/ml yeast tRNA, 0.04% Blocking reagent (Roche #1096176)) and subsequently hybridized overnight at 60°C in hybridization buffer containing *miR-10* LNA probes, labeled and purified as mentioned above, and diluted 1∶50.000. The next day blots were washed 1× at 60°C with hybridization buffer, 1× at 50°C with 2× SSC, 0.1% SDS and 1× at 50°C with 0.1xSSC, 0.1%SDS in order to remove excess probe. Blots were incubated 2× for 5 min. at RT in Maleic Acid buffer (0.1 M Maleic Acid, 150mM NaCl, pH7.5 with NaOH, 0.1% Tween-20) to equilibrate and remove residual SDS. Blots were blocked for 30 min. in blocking buffer (Maleic Acid buffer containing 1% Blocking Reagent (Roche #1096176)) and subsequently incubated for 30 min. in blocking buffer containing 1∶50.000 Anti-Digoxigenin-AP antibody (Roche #093274). Excess antibody was washed away in 4×15 min. washes with Maleic Acid buffer. Blots were subsequently washed 5 minutes in AP-buffer (0.1 M Tris Base, 0.1 M NaCl, pH9.5) and signal was detected on X-Ray film using CDP-star kit (Roche #12041677001) according to manufacturer's instructions with a typical exposure time of 4 hours.

### Retrograde labeling

Anesthetized 5 days old embryos were retrograde labeled by making an incision with a tungsten needle in the spinal cord at the level of the hindgut and injecting 1–5 nl of a concentrated rhodamine-dextran solution. After injection embryos were left to recover for 1–1.5 hrs and fixed in 4% PFA.

## Supporting Information

Figure S1 (0.32 MB PDF)

Figure S2 (0.38 MB PDF)

Figure S3 (0.75 MB PDF)
