# Supplementary material for: MiR-10 Represses HoxB1a and HoxB3a in Zebrafish
Source: PLoS One. 2008 Jan 2;3(1):e1396. doi: 10.1371/journal.pone.0001396 (PMC2148072; doi:10.1371/journal.pone.0001396)
Supplement: Figure S1 — (0.32 MB PDF) [file pone.0001396.s001.pdf]

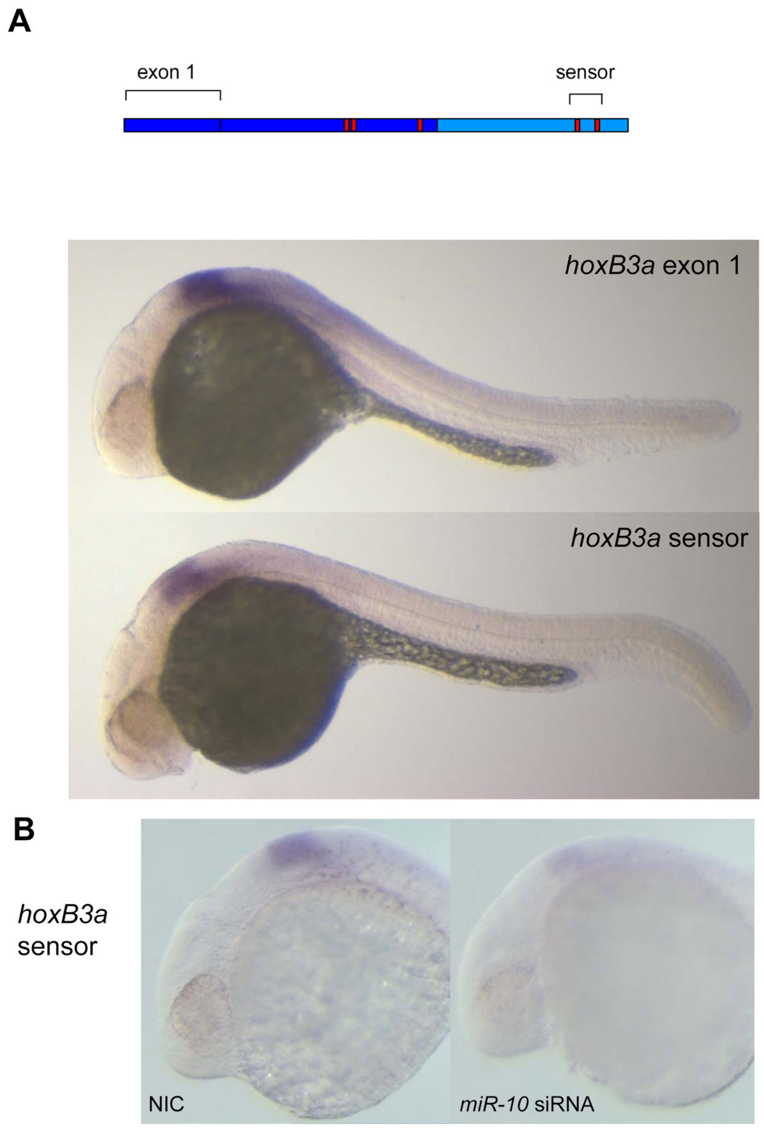

Figure S1) Expression of *HoxB3a* exon1 and *HoxB3a* sensor regions

A) Schematic representation of the *HoxB3a* coding region and 3' UTR. The locations of the regions corresponding to the exon1 probe and the sensor probe are indicated. *In situ* hybridization with the *HoxB3a* exon1 and *HoxB3a* sensor in 24 hpf embryos shows no differences. B) Response of the *HoxB3a* sensor region to *miR-10* siRNA injection in 24 hpf embryos. *In situ* hybridization with the 'sensor' probe shows the same change in expression as *in situ* hybridization with the *HoxB3a* exon1 probe (main figure 4A).
