# Supplementary material for: MiR-10 Represses HoxB1a and HoxB3a in Zebrafish
Source: PLoS One. 2008 Jan 2;3(1):e1396. doi: 10.1371/journal.pone.0001396 (PMC2148072; doi:10.1371/journal.pone.0001396)
Supplement: Figure S2 — (0.38 MB PDF) [file pone.0001396.s002.pdf]

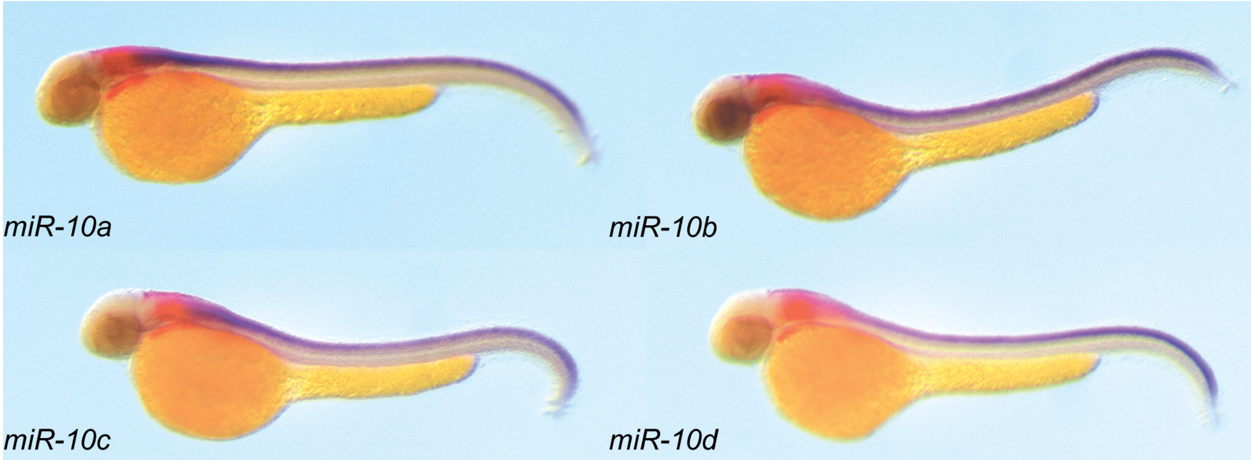

Figure S2) Double *in situ* hybridization of *HoxB3a* with *miR-10a*, *miR-10b*, *miR-10c* and *miR-10d*

Expression of *HoxB3a* exon1 probe (red) and LNA probes for all 4 Zebrafish *miR-10* isoforms (purple) in 72 hpf embryos. All *miR-10* isoforms are expressed posterior from the r5/6 domain of *HoxB3a*. *MiR-10b* and *miR-10d* appear to be expressed slightly more posterior than the *miR-10a* and *miR-10c* isoforms.
