# Supplementary material for: MiR-10 Represses HoxB1a and HoxB3a in Zebrafish
Source: PLoS One. 2008 Jan 2;3(1):e1396. doi: 10.1371/journal.pone.0001396 (PMC2148072; doi:10.1371/journal.pone.0001396)
Supplement: Figure S3 — (0.75 MB PDF) [file pone.0001396.s003.pdf]

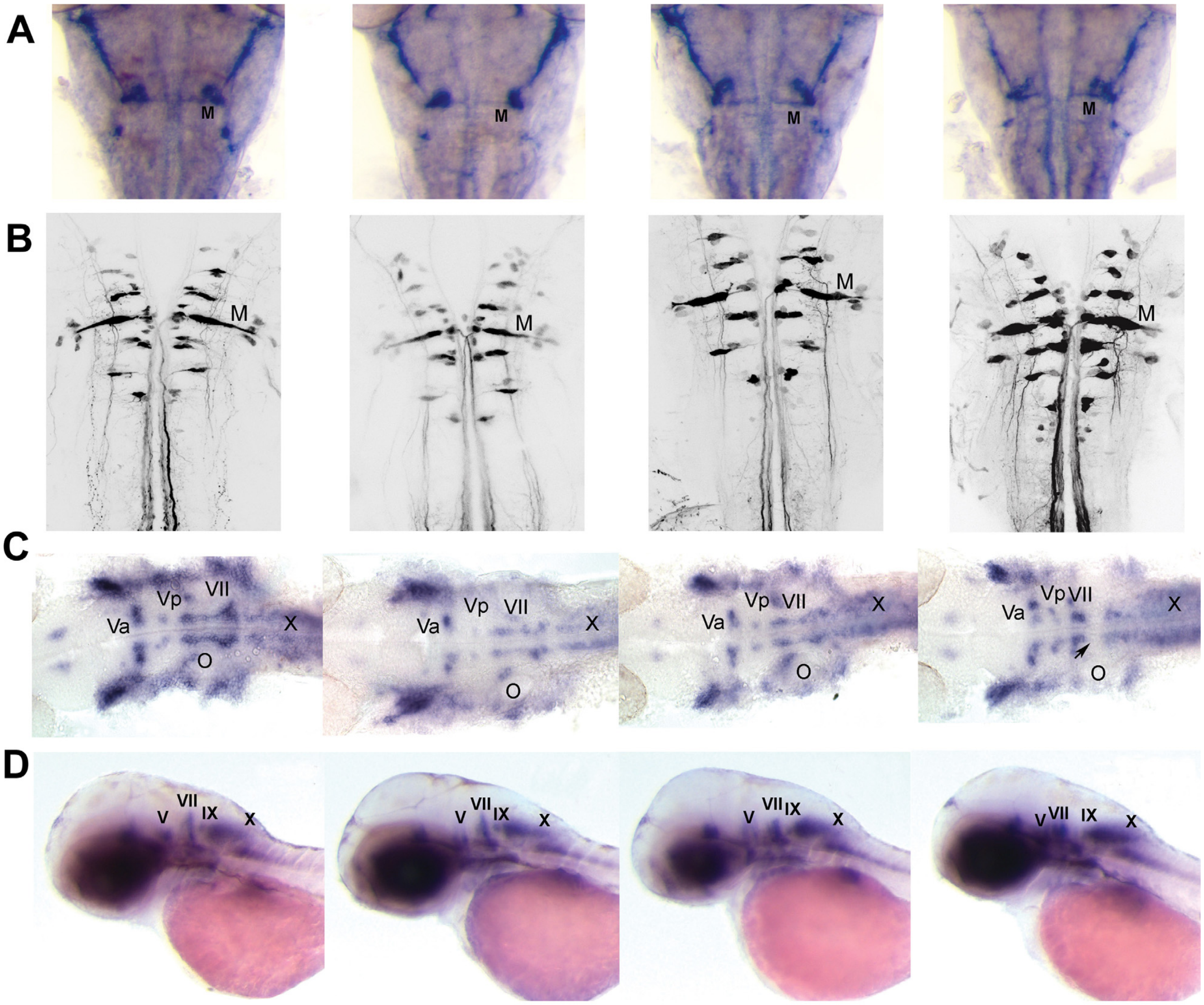

Figure S3) Patterns of primary and secondary hindbrain motorneurons in *miR-10* overexpression and morphant embryos

Column 1; wildtype, column 2; morpholino 1 injected, column 3; morpholino 2 injected, column 4; *miR-10* siRNA injected (also shown in main figure 7)

A) 3A10 neurofilament immunolabeling. Mauthner neurons are present in morphant and overexpression embryos and are indistinguishable from wildtypes. B) Confocal images of hindbrains of 5 day old embryos retrograde labeled embryos. No differences are observed between non injected, morphant or overexpression embryos. C) Flatmounts of 30 hpf embryos *in situ* hybridized with *islet-1*. Patterns of branchiomotorneurons are indicated. Note the failure of the VIIth cranial nerve to migrate into r 5/6. The motorneuron patterns in the morphant embryos are like wildtypes. D) Sideview of 48 hpf embryos *in situ*

hybridized with *islet-1*. In *miR-10* siRNA injected embryos the VIIth nerve is located near the Vth nerve and there is a large gap between the VIIth and the IXth nerve. The patterns in the morphant embryos are like wildtypes.
